# Iodine-123 β-methyl-P-iodophenyl-pentadecanoic Acid (^123^I-BMIPP) Myocardial Scintigraphy for Breast Cancer Patients and Possible Early Signs of Cancer-Therapeutics-Related Cardiac Dysfunction (CTRCD)

**DOI:** 10.3390/jimaging8110296

**Published:** 2022-10-29

**Authors:** Yuko Harada, Kyosuke Shimada, Satoshi John Harada, Tomomi Sato, Yukino Kubota, Miyoko Yamashita

**Affiliations:** 1 Department of Cardiology, Kawasaki Municipal Ida Hospital, Kawasaki 211-0035, Japan; 2 Department of Breast Surgery, Kawasaki Municipal Ida Hospital, Kawasaki 211-0035, Japan; 3 University School of Medicine, Shinanomachi Campus, Tokyo 160-8582, Japan; 4 Department of Palliative Care Medicine, Kawasaki Municipal Ida Hospital, Kawasaki 211-0035, Japan; 5 Department of Radiology, Kawasaki Municipal Ida Hospital, Kawasaki 211-0035, Japan

**Keywords:** breast cancer, echocardiography, ^123^I-BMIPP, myocardial scintigraphy, chemotherapy-related cardiac dysfunction (CTRCD)

## Abstract

(1) Background: The mortality of breast cancer has decreased due to the advancement of cancer therapies. However, more patients are suffering from cancer-therapeutics-related cardiac dysfunction (CTRCD). Diagnostic and treatment guidelines for CTRCD have not been fully established yet. Ultrasound cardiogram (UCG) is the gold standard for diagnosis of CTRCD, but many breast cancer patients cannot undergo UCG due to the surgery wounds or anatomical reasons. The purpose of the study is to evaluate the usefulness of myocardial scintigraphy using Iodine-123 β-methyl-P-iodophenyl-pentadecanoic acid (^123^I-BMIPP) in comparison with UCG. (2) Methods: 100 breast cancer patients who received chemotherapy within 3 years underwent Thallium (^201^Tl) and ^23^I-BMIPP myocardial perfusion and metabolism scintigraphy. The images were visually evaluated by doctors and radiological technologists, and the grade of uptake reduction was scored by Heart Risk View-S software (Nihon Medi-Physics). The scores were deployed in a 17-segment model of the heart. The distribution of the scores were analyzed. (3) Results: Nine patients (9%) could not undergo UCG. No correlation was found between left ventricular ejection fraction (LVEF) and Heart Risk View-S scores of ^201^Tl myocardial perfusion scintigraphy nor those of BMIPP myocardial metabolism scintigraphy. In a 17-segment model of the heart, the scores of the middle rings were higher than for the basal ring. (4) Conclusions: Evaluation by UCG is not possible for some patients. Myocardial scintigraphy cannot serve as a perfect alternative to UCG. However, it will become the preferable second-choice screening test, as it could point out the early stage of CTRCD.

## 1. Introduction

The five-year survival rate of breast cancer exceeds 90% in high-income countries, while breast cancer mortality dropped by 40% between the 1980s and 2020 [1]. Breast cancer mortality decreased due to advancements in cancer therapies. However, more patients are suffering from cancer-therapeutics-related cardiac dysfunction (CTRCD). As breast cancer patients are younger than other cancer patients, CTRCD may adversely affect patient’s quality of life (QOL) and prognosis.

Diagnostic and treatment guidelines for CTRCD have not been established until just recently. The European Society of Cardiology (ESC) Position Paper published in 2016 and the American Heart Association (AHA) statement published in 2019 have only been recently regarded as alternatives to guidelines [2,3]. The worldwide consensus diagnostic criterion for CTRCD is a decrease in the left ventricular ejection fraction (LVEF) of greater than10 percentage points, to a value of less than 53% (normal reference value for two-dimensional echocardiography) [4]. (Note: The first ESC guidelines on onco-cardiology were published concurrent with submission of this paper. See Discussion section).

Ultrasound cardiogram (UCG) is the current initial choice for diagnosis of CTRCD. However, many breast cancer patients are not able to undergo UCG due to surgery wounds, implants, or other anatomical issues in their chest. It is also known that shortfalls in echocardiography are caused by constraints due to dependency on acoustic windows and variable operator skills [5]. Inter-observer variability and image quality are also limitations of UCG [2]. Thus, LVEF is sometimes not available to diagnose CTRCD.

In ESC Position Paper, proposed diagnostic tools for the detection of cardiotoxicity other than UCG are nuclear cardiac imaging (Multigated Radionuclide Angiography, MUGA), cardiac magnetic resonance, and cardiac biomarkers such as Troponin I and benign natriuretic peptide (BNP) [2]. The advantage of MUGA is reproducibility, while its limitations are cumulative radiation exposure as well as its limited structural and functional information beyond LVEF [6]. Some researchers assert that decrease in LVEF is relatively late manifestation of myocardial damage [5]. Therefore, other imaging modalities for evaluating CTRCD are anticipated especially at earlier stage prior to LVEF starting to decease.

Myocardial scintigraphy with Iodine-123 β-methyl-P-iodophenyl-pentadecanoic acid (^123^I-BMIPP) has been widely used in Japan to detect myocardial damage. ^123^I-BMIPP is a tracer for metabolism of fatty acid which is known to account for more than 90% of myocardial energy requirements [7]. ^123^I-BMIPP was introduced in Japan in the early 1990s. It is noted that ^123^I-BMIPP has still not yet been commonly used elsewhere. ^123^I-BMIPP is typically utilized in dual-isotope myocardial scintigraphy with 201-Thallium (^201^Tl) called Tl/BMIPP dual-isotope myocardial scintigraphy.

Tl/BMIPP dual-isotope myocardial scintigraphy has proven to be useful for detecting cardiomyopathies. Decrease of ^201^Tl uptake denotes defect of myocardial perfusion, while decrease of ^123^I-BMIPP uptake denotes decreased metabolic function of myocardium. Therefore, Tl/BMIPP scintigraphy should also be able to diagnose non-ischemic cardiomyopathies, such as Takotsubo cardiomyopathy and drug-induced cardiomyopathy including CTRCD.

The purpose of this study is to evaluate the usefulness of myocardial scintigraphy using ^123^I-BMIPP compared to UCG. As it is well-known that ^201^Tl myocardial perfusion scintigraphy is subjected to artifacts from the breast and liver [8], we purposely focused on ^123^I-BMIPP myocardial metabolism scintigraphy in this research. Since the established resume of myocardial scintigraphy is Tl/BMIPP dual-isotope scintigraphy and not ^123^I-BMIPP alone, both reagents were therefore necessarily utilized.

## 2. Materials and Methods

A total of 114 histologically confirmed breast cancer patients who received chemotherapy for at least 2 months within 3 years were eligible for this study. Those who were not able to communicate, lie on the back, nor remain still were not included. Terminal stage cancer patients were also excluded as they were not eligible for medical benefits for this costly testing. 100 patients were enrolled in this study. All were outpatients of the department of breast surgery at Kawasaki Municipal Ida Hospital, and they provided written consent to be enrolled in this study. Regulatory approval for this study was granted by the research ethics board of Kawasaki Municipal Ida Hospital.

Anti-cancer drugs were given in accordance with the guidelines of the Japanese Breast Cancer Society [9]. All patients underwent UCG prior to chemotherapy and every 6 months during chemotherapy. The most recent LVEF recorded in patients’ charts were collected for this study. For patients whose LVEF fluctuated, the data nearest to the date of scintigraphy were collected.

Tl/BMIPP dual-isotope myocardial scintigraphy was performed on an outpatient basis. Patients fasted for at least 6 h prior to the study, then were injected with 111Mbq of ^123^I-BMIPP (Nihon Medi-Physics Co. Ltd., Tokyo, Japan) intravenously. Single Photon Emission Computed Tomography (SPECT) images were acquired starting from 15 min after tracer injection using a digital gamma camera Symbia E (Canon Medical Systems Corporation, Tochigi, Japan).

**Figure 1:**
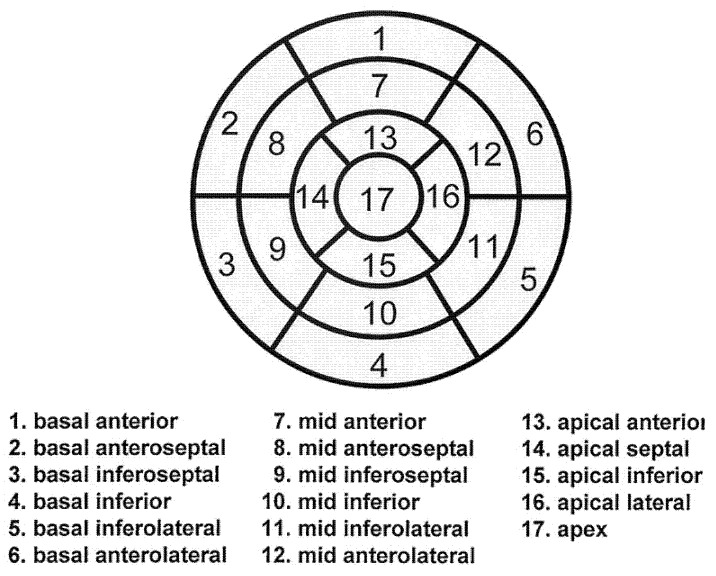
Left ventricular segmentation (17-segment model) [10]. Reprinted/adapted with permission from Ref. [10]. Copyright year 2002, copyright owner American Heart Association, Inc.

Myocardial SPECT images were then analyzed by the automated software program, Heart Risk View-S software (Nihon Medi-Physics Co. Ltd., Tokyo, Japan). The software generated polar maps of the heart from myocardial SPECT data that are divided into 17 segments as recommended by the guideline of the American Heart Association (called 17-segment model, Figure 1) [10]. The software first calculated the mean percentage uptake count for each segment and deployed the percentage in the 17-segment model. These percentages were then compared with the normal ^201^Tl and ^123^I-BMIPP database developed for Japanese patents by the Japanese Society of Nuclear Medicine working group [11,12,13]. The mean percentage uptake count for each segment was then recalculated for the express purpose of eliminating the effect of artifacts; then converted to scores using a five-point scale from normal to absent (0, normal; 4, absent). These recalculated Heart Risk View-S scores were then deployed in 17-segment model as final result. Segments with 60–70%, 50–60%, and 40–50% uptakes were classified as mild, moderate, and severe uptake reductions, and thus scored as 1, 2, and 3, respectively. Segments with uptake less than 40% were scored as 4. Segments with normal uptake were scored as 0.

Since ^201^Tl uptake is easily interfered by artifacts, we focused on ^123^I-BMIPP to evaluate effectiveness of myocardial scintigraphy for detecting CTRCD. The final result by Heart Risk View-s software was 17-segment model polar map with scores 0 to 4, and we used these scores for quantitative comparison. When the polar maps of all patients were visually compared, we noticed the distribution of the scores has a certain pattern. In order to analyze the distribution pattern of the scores, 17-segment model was divided into 4 rings (from outside): basal ring, mid-cavity ring, apical ring, and apex, as nomenclated by AHA [10]. The average scores of each ring were calculated for all patients. Then, each ring was compared with the next ring. The analysis was conducted using the software JMP 16.

## 3. Results

### 3.1. Patients

A total of 100 patients were enrolled in this study. The anti-cancer treatments used for the patients are shown in Table 1.

### 3.2. UCG Evaluation

Measuring LVEF was not possible for 9 patients (9%) due to anatomical issues such as surgery wounds, radiation scars, or implants. 4 patients (4%) were diagnosed with CTRCD according to the criteria of LVEF lower than 53%. However, no symptoms of heart failure were shown. One patient (Case A) was diagnosed with CTRCD 30 months prior to the study; however, her LVEF was normal at enrollment of this study. Another patient (Case B) was diagnosed with myocardial infarction during chemotherapy 18 months prior to this study; however, her LVEF was also normal at enrollment. The details of these 6 patients are described in Table 2.

### 3.3. Myocardial Scintigraphy

**Figure 2:**
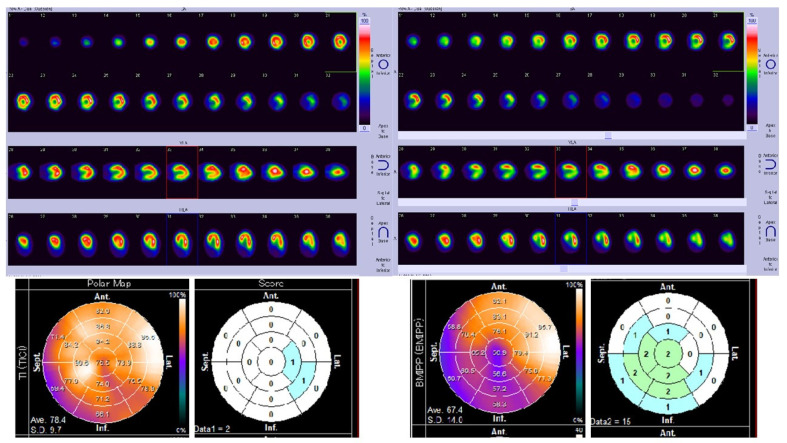
SPECT images, polar maps, and Heart Risk View-S scores of Case E whose LVEF decreased to 48.4%. Left top: SPECT images of ^201^Tl perfusion scintigraphy. Left bottom: polar map and scores of ^201^Tl. Right top: SPECT images of ^123^I-BMIPP metabolism scintigraphy. Right bottom: polar map and scores of ^123^I-BMIPP.

Tl/BMIPP dual-isotope myocardial scintigraphy was performed for all patients. SPECT images, polar map with 17-segment model, and Heart Risk View-S scores deployed in 17-segment model were obtained. As an example of CTRCD, Figure 2 is the data for Case E. Note that ^123^I-BMIPP scores are higher in the middle rings of the 17-segment model. She had no other known cardiac diseases or coronary risk factors.

**Figure 3:**
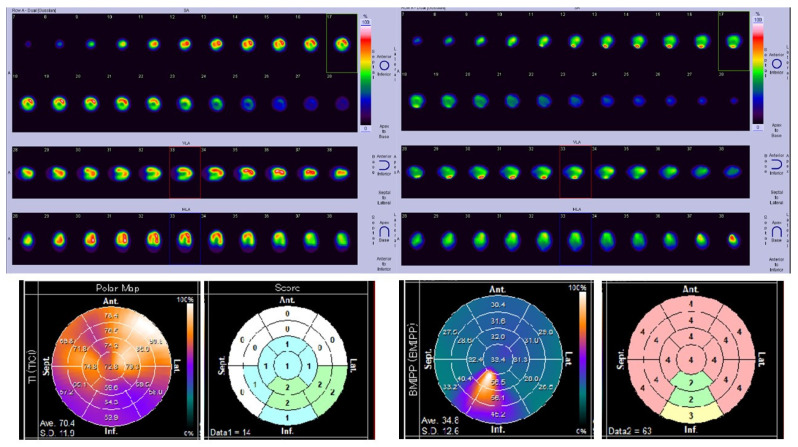
SPECT images, polar maps, and Heart Risk View-S scores for Case G. Left top: SPECT images of ^201^Tl perfusion scintigraphy. Left bottom: polar map and scores of ^201^Tl. Right top: SPECT images of ^123^I-BMIPP metabolism scintigraphy. Right bottom: polar map and scores of ^123^I-BMIPP.

One patient (Case G) revealed remarkably decreased ^123^I-BMIPP uptake, however her LVEF was normal (74.4%). Her chest X-ray, electrocardiogram (ECG), and BNP level were all normal. Her scintigraphy data are shown in Figure 3. Note that ^123^I-BMIPP uptake was remarkably low. She had no other known cardiac diseases or coronary risk factors. She was not diagnosed with CTRCD according to the current diagnostic criteria.

The total Heart Risk View-S scores of ^201^Tl and ^123^I-BMIPP scintigraphy for CTRCD patients are shown in Table 3.

LVEF and the scores did not appear to be correlated. We evaluated the correlation in the next Section 3.4.1.

^201^Tl scores may have been increased by artifacts; therefore, we focused on evaluating ^123^I-BMIPP. For most of the patients, ^123^I-BMIPP scores appeared to be higher in the middle rings as shown in Figure 2 and Figure 3. This is also verified in the next Section 3.4.2.

### 3.4. Mathematical Analysis of Myocardial Scintigraphy

#### 3.4.1. Correlation between LVEF and Isotope Uptake

**Figure 4:**
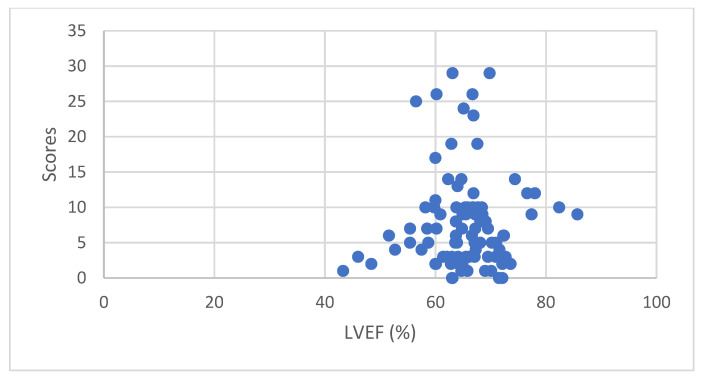
LVEF and Heart Risk View-S scores for ^201^Tl uptake reduction.

Scatter plot of LVEF and Heart Risk View-S scores for ^201^Tl uptake reduction of all 100 patients are shown in Figure 4. Pearson product–moment correlation coefficient was 0.049692 which is an indicator of non-correlation.

**Figure 5:**
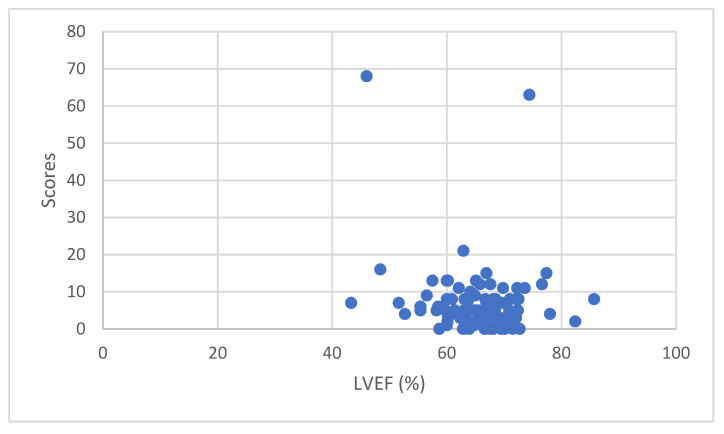
LVEF and Heart Risk View-S scores for ^123^I-BMIPP uptake reduction.

Scatter plot of LVEF and Heart Risk View-S scores for ^123^I-BMIPP uptake reduction of all 100 patients are shown in Figure 5. Pearson product–moment correlation coefficient was −0.14844 which is an indicator of non-correlation. After omitting 2 outliers, Pearson product–moment correlation coefficient was −0.08505 which still indicated non-correlation.

#### 3.4.2. Evaluating the Pattern of ^123^I-BMIPP Uptake Reduction in Patients’ Hearts

All the SPECT images were visually evaluated by cardiologist, breast surgeon, and radiologist. The Heart Risk View-S scores of ^123^I-BMIPP were randomly distributed, and the pattern of score distribution was different from that of ^201^Tl. These revealed that myocardial metabolism decreased diffusely unrelated to myocardial perfusion.

Upon visual evaluation, ^123^I-BMIPP uptake appeared to decrease more in the middle rings of 17-segment model. In order to evaluate the difference between the rings, the average Heart Risk Vies-S scores of each ring (basal, mid-cavity, apical, and apex) were compared and mathematically analyzed using the software JMP 16.

**Figure 6:**
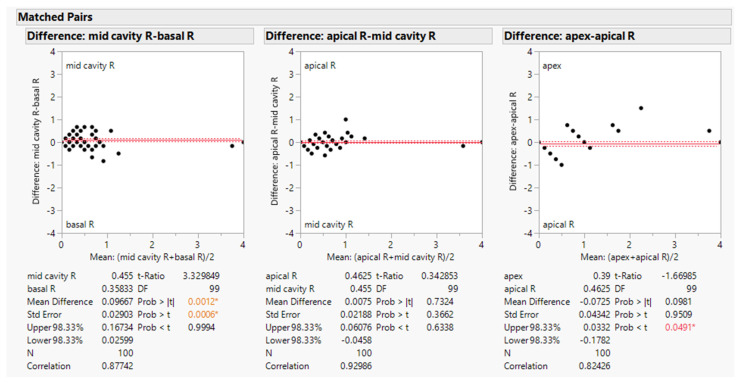
Result of paired two-sided t-test. The *p*-values lower than 0.05 are shown in red color with a star (*).

Equation (1) is for the vertical scale of the scatter plots in Figure 6.

Difference = (average score of the first ring) − (average score of the next ring)(1)

Equation (2) is for the horizontal scale of the scatter plots in Figure 5.

Mean = {(average score of the first ring) + (average score of the second ring)}/2(2)

We conducted paired two-sided t-test (significance level of 5%) for basal ring vs. mid-cavity ring, mid-cavity ring vs. apical ring, and apical ring vs. apex. According to null hypothesis for this t-test, there is not any difference between the average scores of the 2 paired rings. For multiple tests, we utilized Bonferroni correction. As a result, we found a significant difference between the average scores of basal ring and mid-cavity ring (*p* = 0.0012). However, there was not a significant difference between mid-cavity ring and apical ring (*p* = 0.7324), nor between apical ring and apex (*p* = 0.0981). The results are shown in Figure 6.

**Figure 7:**
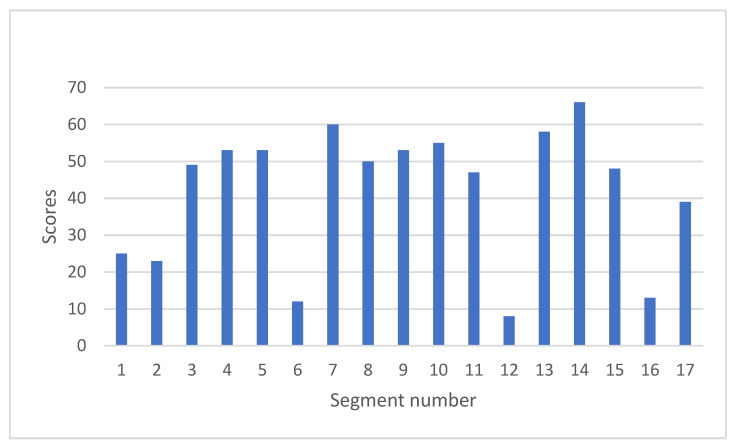
Summed-up Heart Risk View-S scores of ^123^I-BMIPP. All the scores of each segment were summed up for 100 patients. Segment 1 to 6 configures basal ring, segment 7 to 12 configures mid-cavity ring, segment 13 to 16 configures apical ring, and segment 17 is apex, as nomenclated by AHA [10].

Next, the scores of each segment for all 100 patients were summed up as shown in Figure 7. Higher scores denote lower myocardial metabolism, suggesting that the myocardium is more damaged. Segments 6, 12, and 16 revealed lower scores. These are the areas covered by left circumflex artery (LCX) as shown in Figure 1. Middle segments from 7 to 15 revealed higher scores, which is compatible with the result of paired t-test in Figure 6.

## 4. Discussion

Comparison of LVEF and Heart Risk View-S scores did not reveal any correlation. This is not good news for those who anticipated a new imaging modality replacing with UCG. If the scores of ^201^Tl are higher, myocardial perfusion is lower. If the scores of ^123^I-BMIPP are higher, myocardial metabolism is lower. Since these isotope uptakes were not correlated with LVEF, myocardial perfusion and myocardial metabolism were not related to the dynamic function of myocardium in our study. This may be because the cardiac damage of the participants was mild or early stage. In fact, all patients were asymptomatic and 87% of patients showed normal range LVEF (except 4 CTRCD patients and 9 patients whose LVEF were not available). Myocardial metabolism may have lowered prior to decrease in LVEF.

^123^I-BMIPP uptake revealed mismatch with ^201^Tl uptake as expected. Distribution pattern of Heart Risk View-S scores was mathematically analyzed to show how and which part of the heart was damaged by chemotherapy. As a result, middle portions of the heart (mid-cavity ring and apical ring of 17-segment model) were shown to be more damaged. It was a surprising observation that segments 6,12, and 16 revealed lower scores. It may therefore imply that LCX area was spared from damage. Further research is thereby anticipated.

The results of this research constitute observational findings from scintigraphy that have not been heretofore used to evaluate CTRCD. UCG should be the first choice for diagnosis as it is less costly and less invasive than scintigraphy. Alternatively, for patients who cannot undergo UCG, myocardial scintigraphy may become a second choice. Isotopes for scintigraphy must be carefully selected, as each isotope has different characteristics. Nuclear cardiologic techniques visualize pathophysiologic processes at the tissue level; therefore, myocardial injury could be detected at an earlier stage [5]. LVEF represents dynamic function of myocardium, and scintigraphy detects perfusion or metabolic function of myocardium.

^123^I-BMIPP was introduced as a promising new tracer in some reviews [5,6,14]. ^123^I-BMIPP has been used in Japan for over 2 decades, although it has not yet been approved by Food and Drug Administration (FDA) in the U.S. [14]. There are only a few reports regarding ^123^I-BMIPP scintigraphy performed for CTRCD [7,15,16]. Takeishi et al. performed myocardial ^123^I-metaiodobenzylguanidine (MIBG) and ^123^I-BMIPP for 13 patients who received chemotherapy with anthracycline [7]. They used the heart-to-mediastinum ratio (H/M ratio) to compare ^123^I-MIBG and ^123^I-BMIPP, and reported that ^123^I-MIBG could detect myocardial damage at early stage because H/M ratio was lower in the chemotherapy group, while H/M ratio of ^123^I-BMIPP was similar with the control group. However, the H/M ratio is not an established method for evaluating ^123^I-BMIPP scintigraphy. Saito K et al. performed quantitative assessment of the early kinetics of ^123^I-BMIPP by dynamic myocardial SPECT for 26 patients receiving chemotherapy with doxorubicin, and reported that ^123^I-BMIPP uptake was significantly lower after chemotherapy than before hemotherapy [15]. Saito K et al. also performed ^123^I-BMIPP myocardial SPECT for 25 lung cancer patients to conclude that ^123^I-BMIPP is useful for evaluating the cardiotoxicity induced by taxan, because taxan impairs myocardial fatty acid metabolism [16].

There are limitations in this research. First, we could not compare scintigraphy before and after chemotherapy due to medical insurance policy. Therefore, we needed to compare data after chemotherapy with the database in the Heart Risk View-S software. It would have been preferable if scintigraphy was available prior to chemotherapy for each patient. Second, the duration and resume of chemotherapy were different for each patient. Some patients had undergone chemotherapy for years, while others underwent only a few months. It is thus difficult to find patients with exactly the same conditions of chemotherapy at the same time. Third, ^123^I-BMIPP scintigraphy is also interrupted by certain illnesses, such as fatty acid translocase (FAT)/CD36 gene mutation or triglyceride deposit cardiomyovasculopathy (TGCV). FAT/CD36 gene mutation causes a total defect in ^123^I-BMIPP uptake, and its prevalence is 0.47% (33/6970) [17]. Another study reported the prevalence of CD36 deficiency was 0.3–0.5% [18]. Therefore, patients with FAT/CD36 gene mutation should not use ^123^I-BMIPP for CTRCD screening. TGCV is a recently discovered disease which is known to cause decreased ^123^I-BMIPP uptake [19]. TGCV is a rare disease and not much is known about it. In this study, Case G revealed remarkably decreased ^123^I-BMIPP uptake (Figure 3). This patient could have had either FAT/CD36 mutation or TGCV.

Since the early detection and treatment of CTRCD is key to preventing adverse cardiovascular outcomes, new imaging modalities for screening have been anticipated. Biomarkers, cardiovascular magnetic resonance, positron emission tomography, and nuclear imaging have been discussed as potential candidates for screening [20,21]. It is difficult to find a perfect screening test for CTRCD. As ^123^I-BMIPP has shown possible early signs of CTRCD, it may thus be used for one of the screening tests, especially for patients who are unable to undergo UCG.

The world’s first guideline on onco-cardiology was published on 26 August 2022 [22]. However, it does not affect the results of our study. Recommendations for cardiac imaging modalities in patients with cancer have not changed: UCG as the first-line modality, followed by cardiac MRI and MUGA.

## 5. Conclusions

^123^I-BMIPP myocardial metabolism scintigraphy revealed possible early signs of CTRCD for breast cancer patients. ^123^I-BMIPP uptake has shown to decrease in the middle rings of the 17-segment heart model in this study. FAT/CD36 gene mutation and TGCV may be contraindications for ^123^I-BMIPP scintigraphy screening for CTRCD. For breast cancer patients who are unable to undergo UCG, ^123^I-BMIPP scintigraphy will become the preferable second-choice screening test for CTRCD.

## Figures and Tables

**Table 1:** Anti-cancer treatments used for patients.

Treatment Therapy	Percentage (%)
Trastuzumab	49
Pertuzumab	21
Trastuzumab Emtansine (T-DM1)	13
Epirubicin	62
Paclitaxel (PTX)	90
Radiation therapy	36

**Table 2:** Patients diagnosed with CTRCD.

Patient	Age	LVEF at Onset (%)	Latest LVEF (%)	Anti-Cancer Drugs ^1^
Case A	67	46.6	59.8	Epirubicin, PTX
Case B	66	48	78	Epirubicin, PTX, Trastuzumab, Pertuzumab, T-DM1
Case C	76	43.3	43.3	Epirubicin, PTX
Case D	72	46	46	Epirubicin, PTX, Trastuzumab, Pertuzumab
Case E	78	48.4	48.4	PTX, Trastuzumab, T-DM1
Case F	60	51.6	51.6	PTX, Trastuzumab

^1^ Only the main drugs are listed here.

**Table 3:** Patients diagnosed with CTRCD.

Patient	Age	LVEF at Onset (%)	Latest LVEF (%)	^201^Tl Scores ^1^	^123^I-BMIPP Scores ^1^	Anti-Cancer Drugs ^2^
Case A	67	46.6	59.8	10	5	Epirubicin, PTX
Case B	66	48	78	12	4	Epirubicin, PTX, Trastuzumab, Pertuzumab, T-DM1
Case C	76	43.3	43.3	1	7	Epirubicin, PTX
Case D	72	46	46	3	68	Epirubicin, PTX, Trastuzumab, Pertuzumab
Case E	78	48.4	48.4	2	16	PTX, Trastuzumab, T-DM1
Case F	60	51.6	51.6	6	7	PTX, Trastuzumab

^1^ Denotes Heart Risk View-S scores. ^2^ Only the main drugs are listed here.

## Data Availability

Not applicable.
